# Histological characteristics of ligament healing after bio-enhanced repair of the transected goat ACL

**DOI:** 10.1186/s40634-015-0021-5

**Published:** 2015-02-28

**Authors:** D Tan Nguyen, Sietske Dellbrügge, Paul P Tak, Savio L-Y Woo, Leendert Blankevoort, Niek C van Dijk

**Affiliations:** Department of Orthopedic Surgery, Orthopaedic Research Center Amsterdam, Academic Medical Center, University of Amsterdam, P.O. Box 22660, 1100 DD Amsterdam, The Netherlands; Division of Clinical Immunology and Rheumatology, Academic Medical Center, University of Amsterdam, Amsterdam, The Netherlands; GlaxoSmithKline, Stevenage, U.K. and University of Cambridge, Cambridge, UK; Department of Bioengineering, Musculoskeletal Research Center, University of Pittsburgh, Pittsburgh, USA

**Keywords:** Anterior cruciate ligament, Healing, Bio-enhanced ACL repair, Primary repair, Small intestine submucosa

## Abstract

**Background:**

Recently, healing of a ruptured anterior cruciate ligament (ACL) is reconsidered. In a previous study, we have shown that the transected ACL can heal after treatment with the triple X locking suture alone or combined with small intestine submucosa (SIS). The first research question of this study was whether the healing ACLs in both groups show histological characteristics that are typical for ligament healing. Secondly, did the combined treatment with SIS lead to improved histological healing, in terms of the morphology of the fibrous synovial layer, the extracellular matrix (ECM), collagen fiber orientation, cellularity, ratio of myofibroblasts, and collagen type 3 staining. The hypothesis was that SIS enhances the healing by the scaffolding effect, endogenous growth factors, and chemoattractants.

**Methods:**

In the Suture group, the left ACL was transected and sutured with the triple X locking suture repair technique. In the Suture-SIS group, the left ACL underwent the same procedure with the addition of SIS. The right ACL served as internal control. Standard histology and immunostaining of α-smooth muscle actin (SMA) and collagen type 3 were used.

**Results:**

Microscopy showed that the fibrous synovial layer around the ACL was reestablished in both groups. The collagen fibers in the Suture-SIS group stained denser, were more compactly arranged, and the ECM contained fewer voids and fat vacuoles. Neovasculature running between the collagen fibers was observed in both experimental groups. Collagen type 3 stained less in the Suture-SIS group. The cellularity in the Suture group, Suture-SIS group and Control was 1265 ± 1034 per mm^2^, 954 ± 378 per mm^2^, 254 ± 92, respectively; 49%, 26% and 20% of the cells stain positive for α-SMA, respectively.

**Conclusion:**

The healing ACL in both treated groups showed histological characteristics which are comparable to the spontaneously healing medial collateral ligament and showed that the ACL has a similar intrinsic healing response. Though, no definitive conclusions on the beneficial effects of the SIS scaffold on the healing process can be made.

## Background

The anterior cruciate ligament (ACL) of the knee joint is frequently ruptured and may often require reconstruction with autologous tendon grafts to treat chronic knee instability (Beynnon et al. 2005; Woo et al. 2006). The clinical and functional outcome of ACL reconstruction is generally satisfactory, allowing the majority of the patient population to return to work and a part to return to pre-injury level sports activity. However, ACL reconstruction does not fully restore the function of the intact ACL. Additionally, there are several drawbacks (Busam et al. 2008; Drogset et al. 2010; von Porat et al. 2004). Though, it is commonly believed that the ACL does not have a healing response and cannot heal, some researchers are re-exploring methods to repair the ACL in the acute phase (Fisher et al. 2012; Fleming et al. 2009; Murray et al. 2007; Nguyen et al. 2013; Kohl et al. 2013). In a previous study, our research group reported that the treatment of the transected ACL with a new suture repair technique in combination with the small intestinal submucosa (SIS) bioscaffold lead to healing in a goat model (Nguyen et al. 2013). The ACLs in both experimental groups were healing and continuous. Biomechanical testing showed that the repaired ACLs contributed to the knee function (Nguyen et al. 2013). The total AP translation of the repaired ACL was 290% to 440% of the intact control under 67 N anterior and posterior tibial load (AP load). The normalized stiffness of the healing ACLs was about half of the control ACLs. The ACLs were retained for this study with the aim to histologically investigate whether the healing ACL has histologically characteristics as the healing medial collateral ligament (MCL). The comparison with the MCL was made as the MCL is regarded as the knee ligament that has a healing response and that can heal spontaneously. As such, the first research question of this study was whether the ACLs treated with the triple X suture alone or combined with small intestine submucosa (SIS) resemble the histological healing characteristics as observed in the healing MCL. Secondly, does the combined treatment with SIS leads to improved histological healing characteristics, in terms of the morphology of the fibrous synovial layer, the extracellular matrix (ECM), collagen fiber orientation, cellularity, ratio of myofibroblasts over total cell count, and collagen type 3 staining. These parameters provide a general evaluation of the healing process in ligaments. Myofibroblasts have shown to play an important role in the healing and remodeling of MCL, with an initial increase in density after injury and steadily normalization during the remodeling phase. Myofibroblasts have also been shown in the injured but non-healing ACL, though the density remained low (<1.5%) (Menetrey et al. 2011). SIS bioscaffold is mainly composed of collagen type 1 and contains endogenous growth factors such as fibroblast growth factors (FGF) and TGF-β, as well as other chemoattractants which enhances the healing (Hodde et al. 1996; Hodde et al. 2005; Voytik-Harbin et al. 1997). Several studies have shown that SIS can act as a provisional scaffold to promote cell migration and to enhance revascularization and repair (Gilbert et al. 2007; Liang et al. 2008; Zantop et al. 2006; Liang et al. 2011; Raeder et al. 2002). It was thus hypothesized that SIS enhances the healing of the ACL and that the SIS-treated ACL is closer to the normal ACL histologically, i.c. compacter ECM, less voids, more cells, less myofibroblasts and less collagen type 3 staining.

## Methods

The goat ACLs from the Suture group (n = 4), Suture-SIS group (n = 4), and their respective intact control group (n = 8) were retained from a previous study (Nguyen et al. 2013). Initially, there were five samples in the Suture group; however due to an embedding error and consequently cutting errors one sample was lost for histological analysis. For a detailed description of the animal protocol and surgery, which was approved by the institutional Animal Ethics Committee at the University of Amsterdam, see Nguyen et al., (Nguyen et al. 2013). Briefly, in the left goat knee, a medial parapatellar incision was made to expose the ACL. The ACL was hooked and transected in its midsubstance. In the Suture repair group, the suture repair was performed using absorbable Vicryl 2–0 sutures, using a previously described triple X locking suture technique. Due to its locking configuration, the suture tightens around the collagen fibers when tensile forces are applied, thus providing approximation under tension, greater ultimate tensile strength, and resistance to gapping (Becker and Davidoff 1977; Hatanaka et al. 2000). Subsequently, the medial arthrotomy was closed in separate layers. In the Suture-SIS group, the same procedures were performed as in the Suture group with the addition of porcine derived SIS (Cook Biotech Inc., West Lafayette, IN, USA). Six small pieces of SIS (2 mm x 2 mm x 200 μm) were loosely placed within the midsubstance of the injury site. A hydrated sheet of SIS (5 cm x 2,5 cm x 200 μm) was wrapped around the injury site and affixed with Vicryl 6–0 to the ACL. The ACL in the right hind limb was unoperated in both groups and served as internal intact control. Postoperatively, the animals were allowed full weight bearing immediately after the operation without any external bracing of the operated limb. Free cage activity and later a pasture, food and water was *ad libitum*. At twelve weeks post-surgery, all animals were euthanized and the hind limbs were immediately stored at −20°C until biomechanical testing. The ACLs were kept intact during biomechanical testing and were not tested to failure. The whole ACL was removed from the femur-ACL-tibia complex by cutting the whole ACL from its tibial and femoral bone insertion. The whole ACL was embedded in Tissue-Tek O.C.T. compound (Sakura Finetek, Japan), frozen, and stored at −80°C.

### Histology

Five-micrometer thick sections were cut in a cryostat microtome and mounted on glass slides (Star Frost adhesive slides, Knittelgläser, Germany), and stored at −80°C until staining. The cryosections was made by SD and number coded. The staining and analyses were done by DTN and MdB. Standard Haematoxylin & Eosin (H&E) was performed to grossly evaluate the fibrous synovial membrane, the ECM, and cell morphology. Lendrum Masson’s trichrome staining was performed to specifically stain the collagen and to evaluate the collagen fiber orientation. The sections were stained sequentially in celestine blue, haematoxiline, Ponceau de Xylidine and tartrazine. The collagen was stained in yellow, the cytoplasm in red and the nuclei in blue. The slides were observed under a light microscope and pictures were taken from the midsubstance at the healing areas with a low magnification (10X) to show the whole ACL and with higher magnifications (100X, 200X and 400X).

### Immunohistochemical staining

An AEC (3-Amino-9-ethylcarbazole) labeling immunohistochemical methodology was used with the following antibodies: goat monoclonal mouse anti-human alpha-smooth muscle actin (α-SMA) antibody (Sigma-Aldrich, St. Louis, MO) as a marker for myofibroblasts and monoclonal mouse anti-human collagen type 3 antibody (Abcam, Cambridge, UK) to identify collagen type 3. Briefly, the sections were fixed in 100% acetone for 10 min at room temperature and washed with phosphate-buffered saline (PBS), three times for 5 minutes. Endogenous peroxidase activity was blocked using peroxidase 3% for 30 minutes at room temperature. The sections were rinsed once in PBS and 10% normal goat serum, 1% Bovine Serum Albumin (BSA) in PBS was applied for 1 hour at room temperature as a blocking agent. The monoclonal α-SMA antibody in 5% normal goat serum, 1% BSA in PBS (5 μg/ml) was applied and incubated overnight at 4°C on a shaker. Collagen type 3 was stained with monoclonal mouse collagen type 3 antibody in 5% normal goat serum, 1% BSA in PBS (0.5 μg/ml). Subsequently the sections were washed in PBS three times for 5 minutes each. The secondary antibody, a rabbit anti-mouse immunoglobulin (IgG) horseradish conjugate (HRP) (Abcam, Cambridge, UK), was applied to the sections 1 μg/ml for 60 minutes at room temperature. After a final rinse with PBS, HRP activity was visualized as a brown-red color by incubation with AEC (Sigma-Aldrich). Sections were counterstained with Mayer’s haematoxylin and mounted in Kaiser’s glycerol gelatin (Merck). Appropriate isotype controls were assessed in all experimental series. Positive controls for α-SMA and collagen type 3 were goat artery vessels and goat skin, respectively.

### Digital image analysis

Quantification of α-SMA expressing cells in the healing tissue was performed with digital image analysis (DIA), as previously described (van der Hall et al. 2007). In brief, for each acquisition session the microscope, camera, and computer were calibrated according to a standardized procedure. Settings are recorded and stored and used for the entire session. After immunohistochemical staining, three representative regions of 1.45 x 1.45 mm in each section are identified at low power magnification (without capillaries, fat voids and artifacts) and separated in 18 consecutive high power fields (HPF). The18 HPF images are analyzed by computer-assisted image analysis using a Syndia algorithm on a Qwin-based analysis system (Leica, Cambridge, UK) (Haringman et al. 2005). The software identified positive cells by combining two masks where areas of a nucleus surrounded by a red-brown staining are identified as positive cells, and isolated blue (nuclei without staining) or red are ignored. Positive staining of α-smooth muscle actin was expressed as number of positive cells/mm^2^.

Two sections per sample stained for collagen type 3 (n = 16) are semi-quantitatively analyzed by DTN and MdB with a standard binocular light microscope (Olympus) at 200X magnification. The expression of collagen type 3 was scored on a 5-point scale (range 0–4), as previously described (Tak et al. 1995). A score of 0 represented no expression, while a score of 4 represented abundant expression of collagen type 3 within the healing tissue. Differences between the two observers are resolved by consensus.

### Statistical test

A paired or unpaired Student’s t-test was used for statistical analysis, where appropriate. A probability of p < 0.05 was considered statistically significant.

## Results

### Gross morphology

**Figure 1:**
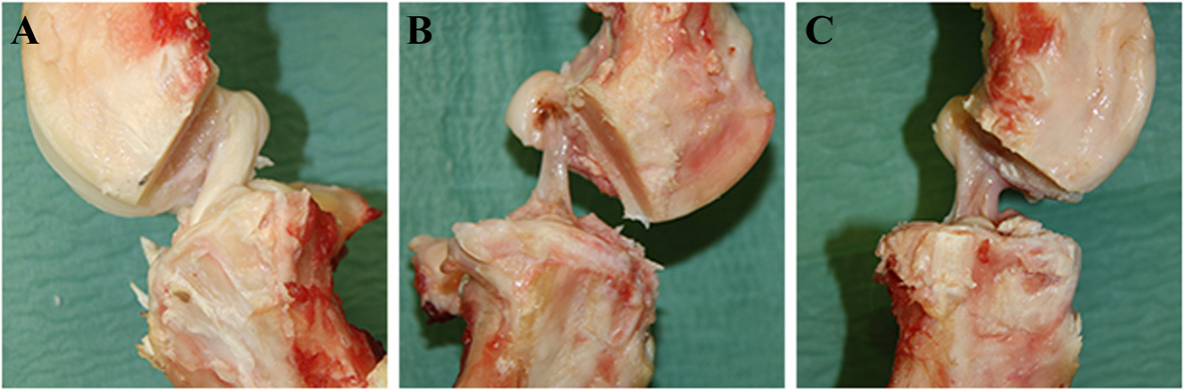
**Gross morphology. A)** Medial side view of a normal ACL. **B)** A healing ACL after suture repair only. **C)** A healing ACL after suture repair and SIS.

The ACLs in both experimental groups are healing and continuous. The transection site cannot be recognized. The Vicryl sutures and SIS are fully resorbed. In the Suture-SIS group, the ACL appears more opaque and denser than the Suture group (Figure 1). The cross-sectional areas of the healed ACLs in the Suture group and Suture-SIS group as reported previously are 35% and 50% of the intact control (Nguyen et al. 2013).

### Fibrous synovial layer

**Figure 2:**
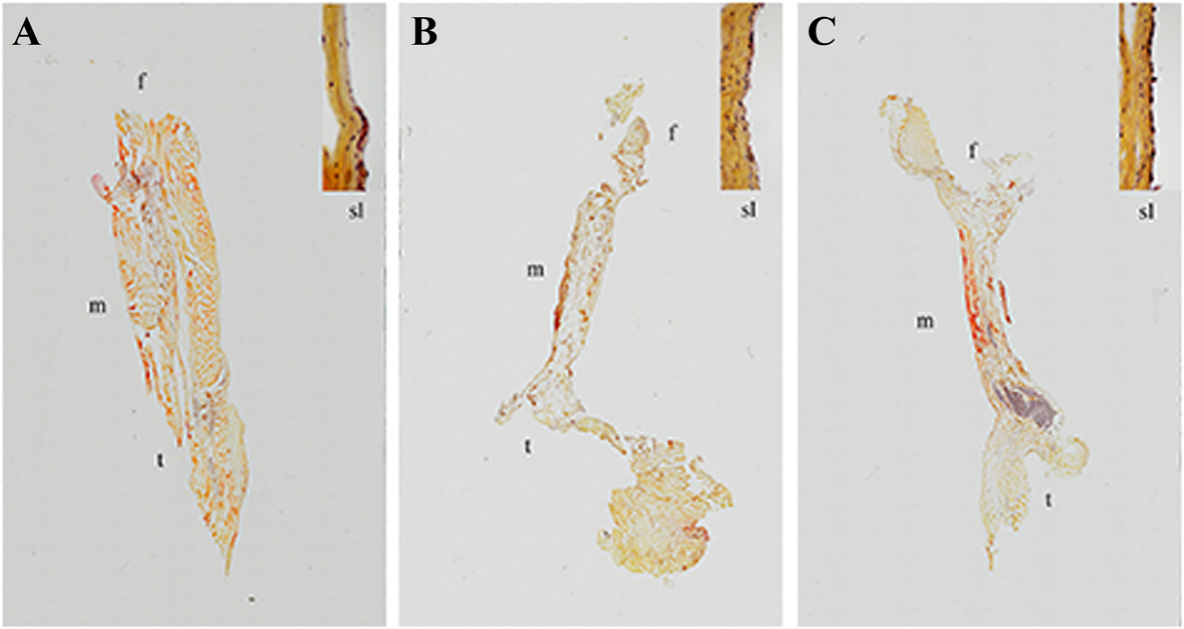
**Histologic sections of a normal ACL (A), sutured ACL (B) and sutured ACL with SIS (C) with magnification of subintimal synovial layer at the top right (×100).** The sections are stained with Lendrum Masson’s trichrome; the collagen fibers are stained in yellow, the cytoplasm in red and the nuclei in blue.

Microscopy shows that the surface of the ACL in both the two experimental groups and the intact control ACLs were covered by a layer of cells and formed the fibrous synovial layer (Figure 2). The sub-intimal synovial layer can be observed along the whole length of the ACL without any disruption.

### Extracellular matrix

**Figure 3:**
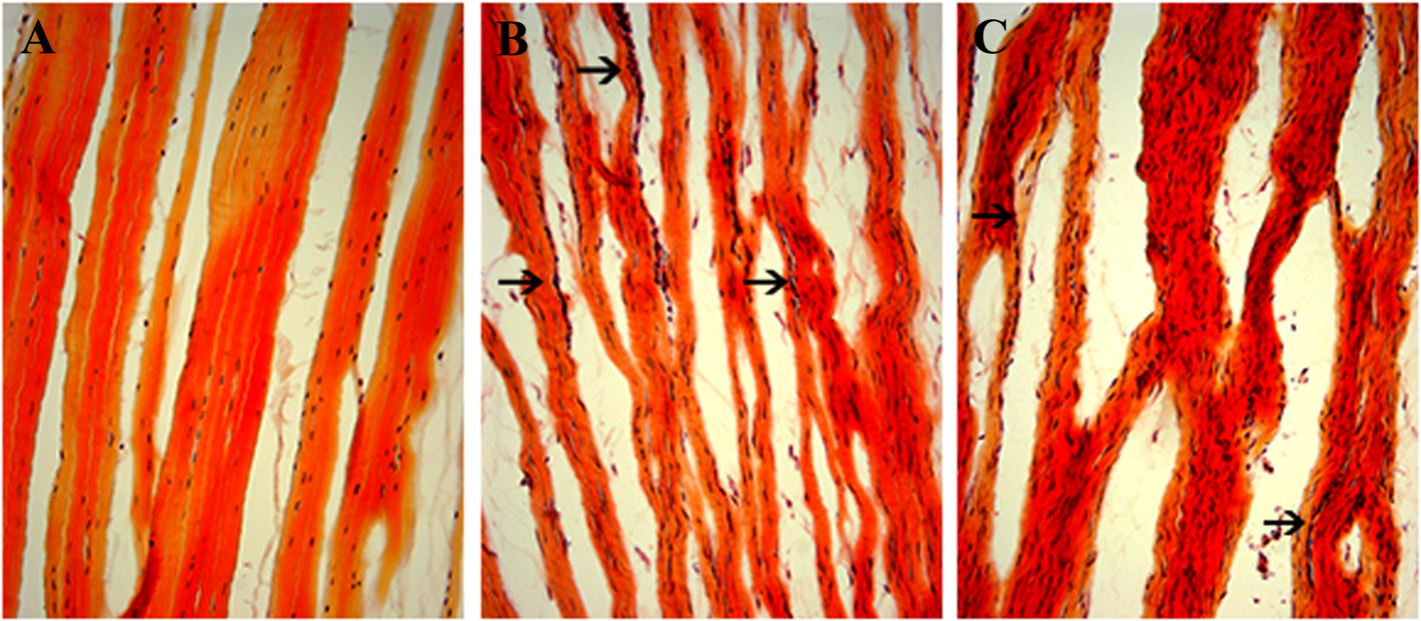
**Histologic sections of a normal ACL (A), sutured ACL (B) and sutured ACL with SIS (C).** The sections are stained with Lendrum Masson’s trichrome; the collagen fibers are stained in yellow, the cytoplasm in red and the nuclei in blue. The ECM of both the Suture and Suture-SIS group have aligned collagen fibers, the collagen fibers in the Suture-SIS group stained denser, more compact arranged and contained less voids and less fat vacuoles. Neovasculature running parallel between the collagen fibers could be observed in both experimental groups. ×100.

**Figure 4:**
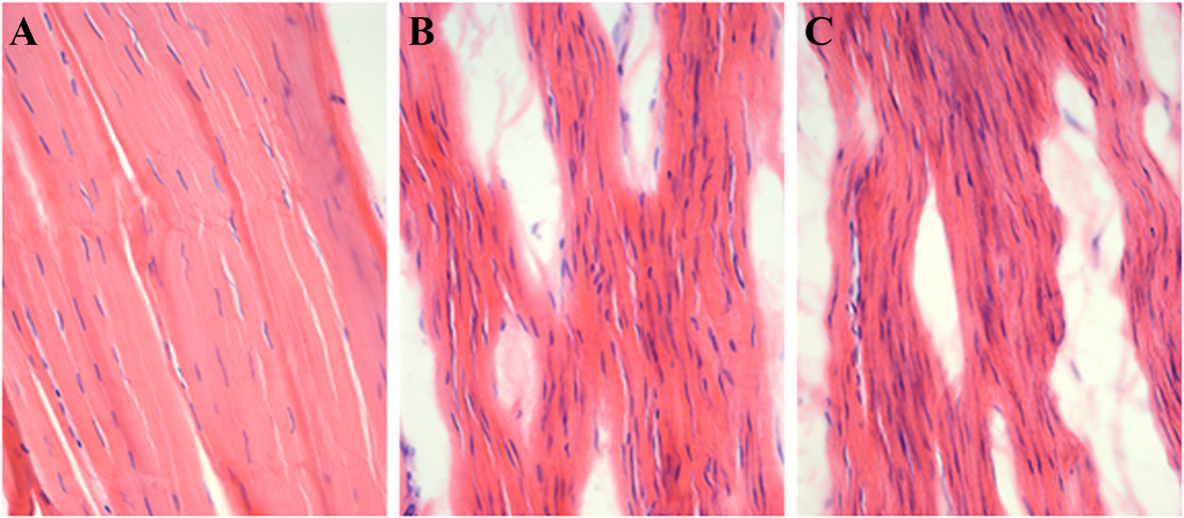
**Histologic sections of a normal ACL (A), sutured ACL (B) and sutured ACL with SIS (C).** The sections are stained with H&E. Note that the tissue is hypercellular in both the sutured and Suture & SIS ACL and that the nuclei are elongated while in the intact ACL the nuclei are rod-like shaped. ×200.

**Figure 5:**
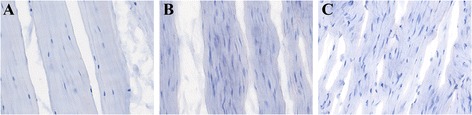
**Immunohistologic sections of a normal ACL (A), sutured ACL (B) and sutured ACL with SIS (C).** B and C show moderate red-brown staining of collagen type 3. (×400).

H&E and Lendrum’s Masson’s trichrome shows that the ECM in the normal ACL was densely packed with aligned collagen fibers and a crimp pattern. No apparent capillaries, voids or lipid vacuoles can be observed (Figures 3A and 4A). Immunostaining of collagen type 3 shows that there was limited staining, scoring 1 (Figure 5A). In the Suture group the collagen fibers are aligned but loosely packed and with patches of more compact fibers but with no crimp pattern at the midsubstance (Figures 3B and 4B). Voids and lipid vacuoles can be observed. Many capillaries can be observed between the fibers, and in some slides the capillaries can be traced through the ACL. Collagen type 3 was present with a score of 2 (Figure 5B). In the Suture-SIS group the collagen fibers were aligned and more compact organized than the Suture group, but with no crimp pattern at the midsubstance (Figures 3C and 4C). Voids and lipid vacuoles can also be observed but was less present than in the Suture group. Many capillaries can be observed between the fibers, and in some slides the capillaries can also be traced through the ACL. Collagen type 3 is present with a score of 2 (Figure 5C). In all groups, the ACLs were vital and there were no necrotic areas.

### Fibroblast morphology, cell and myofibroblast density

**Figure 6:**
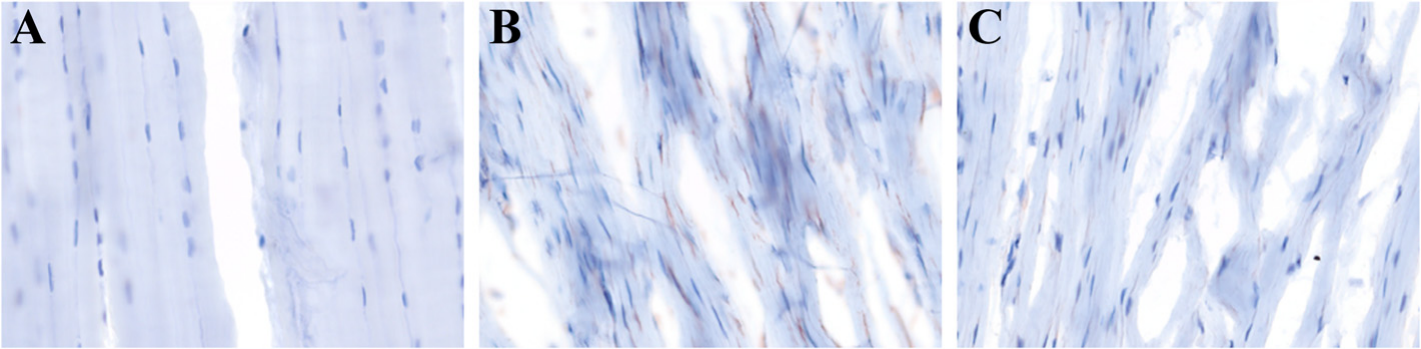
**Immunohistologic sections of a normal ACL (A), sutured ACL (B) and sutured ACL with SIS (C).** Example of a digital image analysis of α-smooth muscle actin expressing cells. Nuclei stained in blue and α-smooth muscle actin stained in red-brown, identifying α- smooth muscle actin expressing cells. (×400).

In the control groups, normal nucleus morphology and cell distribution can be observed within the ACL. Generally, ovoid fibroblast nuclei were located at the proximal ACL, rod-like fibroblast nuclei were located in the midsubstance (Figures 4A and 6A). Round to ovoid shaped nuclei were located at the distal part. Some “chrondroblasts” were observed at the distal part of the ACL. Quantification of the cells with the DIA shows that the cell density in the midsubstance of the Suture control group and Suture-SIS control group was 254 ± 92 cells/mm^2^ and 204 ± 93 cells/mm^2^, respectively. The ratio of α-SMA positive cells in the control groups was 19% and 20%, respectively (Table 1).

**Table 1:** **Total cell density and myofibroblast cell density in the samples of healing ACL tissue (mean ± sd)**

	**Suture**		**Suture-SIS**	
	**Healing**	**Control**	**Healing**	**Control**
Number of cells per mm^2^	1265 ± 1034	254 ± 92	954 ± 378*	204 ± 93*
Number of myofibroblast per mm^2^	642 ± 564	52 ± 22	266 ± 229	36 ± 15
Ratio myofibroblast/total cell density	49%	20%	26%	19%

The difference between Suture-SIS and Control was statistically significant (*paired Student t-test, p < 0.05). There was no statistical difference between the Suture and Suture-SIS group.

In the Suture group, nucleus morphology and cell distribution were different than in the control group. Limited variation in nucleus morphology was observed. Generally, the nuclei were elongated with more of a spindle shape and were scattered over the whole ACL. The cell density was 1265 ± 1034 and the ratio of α-SMA positive cells over total cell count was 49% (Table 1). The difference in cellularity and the difference in the ratio of the number of α-SMA positive cells over the total number of cells between the Suture group and its control group was statistically not significant (p > 0.05).

In the Suture-SIS group, nucleus morphology and cell distribution also differ from the control group and were comparable to the Suture group (Figures 4C and 6C). The healing ACLs in the Suture-SIS group were hypercellular with a cell density of 954 ± 378 cells/mm^2^. The difference in cellularity between the Suture-SIS group and its control group was statistically significant (p = 0.048) (Table 1). The ratio of α-SMA positive cells of the Suture-SIS group was 26% and neared the control group (Table 1). Comparing the cellularity and the ratio of α-SMA positive between the Suture group and the Suture-SIS group, shows that there were no statistically significant differences.

## Discussion

Previously, our research group has shown that a new suture technique alone or combined with SIS bioscaffold resulted in healing of the transected goat ACL (Nguyen et al. 2013). Knee laxity and ligament stiffness did not return to normal values. The difference between the Suture, Suture-SIS and intact control group was 53% and 51% respectively. However, the outcome was close to previously reported results of bone-patellar tendon-bone ACL reconstructions in goats (Spindler et al. 2009; Abramowitch et al. 2003). The results of this study shows that the healing ACL in the Suture group displayed typical histological characteristics like those observed in spontaneously-healing ligaments, such as the medial collateral ligament (MCL). The MCL’s histological characteristics include, for example, disorganized and less compact organized collagen fibers, increased neovascularization, voids (e.g. lipid vacuoles), increased number of myofibroblasts and elevated content of collagen type 3 (Frank et al. 1983; Faryniarz et al. 1996; Amiel et al. 1987; Menetrey et al. 2011). Therefore, it can be concluded that the ACL has a healing response. Adding SIS to the Suture repair seems to biologically enhance the healing as the collagen fibers were more compactly arranged, contain less collagen type 3, and fewer voids and fat vacuoles. The ratio of myofibroblasts to total cell count was more normal. Although this study was not designed to elucidate the mechanism behind the effects of SIS on the healing of the ACL, it was likely that the same mechanisms were involved as previously reported. In previous MCL healing studies, it was also shown that SIS acted as a provisional scaffold to promote cell migration and to enhance revascularization and repair (Gilbert et al. 2007; Liang et al. 2008; Zantop et al. 2006; Liang et al. 2011; Raeder et al. 2002). SIS treatment resulted in a greater alignment of collagen fibers and cells. Ultrastructurally, the SIS-treated group had larger collagen fibrils, and the gene expression of collagen type V, decorin, biglycan, and lumican in the SIS-treated group were significantly down-regulated, correlating with the improved morphological characteristics and mechanical properties (Liang et al. 2008). Other studies have indicated that the hypocellularity of the normal ACL might be a limiting factor leading to the failure of healing of the ACL. However, in this study it was shown that the ACL in the Suture-SIS group can become hypercellular. Thus, hypercellularity may be a positive indicator of ACL healing. Additionally, the fibroblasts in the healing ACLs were not rodlike-shaped but elongated and arranged in an aligned network. This reticular arrangement, hypercellularity, and phenotype may facilitate cell-to-cell communication and aligned deposition of newly formed collagen (Nguyen et al. 2009; Wang et al. 2003; Birk and Zycband 1994). The cells that contribute to the healing may originate from the proliferating internal ligament fibroblasts or may be bone marrow-derived or a combination of these (Zantop et al. 2006; Badylak et al. 2001; Beattie et al. 2009). The ratio of myofibroblasts in the SIS treated ACLs neared that of the normal ACL. Whether this normalization represents a good healing response cannot be answered by this study. It may be a positive indicator, as a decrease in myofibroblasts may indicate that the fibroblasts within the SIS-treated ACLs did not need to maintain the contractile phenotype as they experienced less strain within a larger cross-sectional area. Regarding the vasculature, in both experimental groups, neovasculature through the healing ACL was clearly present. In summary, all these histological observations indicate that the whole ACL was involved in the healing of the injury site.

While this study provides some new insights into the histological morphology of the healing ACLs after Suture and Suture-SIS treatment, there were several limitations. The ACLs were not directly frozen into −80°C which may have influenced and reduced the immunohistological stainings of collagen type 3. Furthermore, the evaluation of the H&E and Lendrum’s Masson’s trichrome was qualitative, though standard histology is commonly used as it provides a representative impression. Finally, the healing ACLs were examined at one time point which does not allow the evaluation of the healing process over time and there was a limited sample size.

Comparing the histological observations of the healing ACL to the ligamentization of tendon grafts in ACL reconstruction, it was shown that the healing ACLs were vital, while it has been reported that necrotic areas within the core of tendon grafts can still be observed during the ligamentization process at 12 weeks (Kondo et al. 2012).

Future studies will include rehabilitation protocols in order to prevent excessive loads on the ACL for potentially a better healing, by e.g. (short-term) casting or internal augmentation devices (Kohl et al. 2013; Fisher et al. 2012).

## Conclusions

In conclusion, the healing ACL in both treated groups showed histological characteristics which are comparable to the spontaneously healing medial collateral ligament and shows that the ACL has a similar intrinsic healing response. The addition of SIS appears to biologically enhance the healing process as shown by a continuous fibrous synovial layer, more compact ECM, fewer voids, more aligned collagen fibers, less collagen type 3, and a decreased ratio of myofibroblasts. Though, no definitive conclusions on the beneficial effects of the SIS scaffold on the healing process can be made.
